# Identification of Special AT-Rich Sequence Binding Protein 1 as a Novel Tumor Antigen Recognized by CD8^+^ T Cells: Implication for Cancer Immunotherapy

**DOI:** 10.1371/journal.pone.0056730

**Published:** 2013-02-21

**Authors:** Mingjun Wang, Bingnan Yin, Satoko Matsueda, Lijuan Deng, Ying Li, Wei Zhao, Jia Zou, Qingtian Li, Christopher Loo, Rong-Fu Wang, Helen Y. Wang

**Affiliations:** 1 Center for Inflammation and Epigenetics, The Methodist Hospital Research Institute, Houston, Texas, United States of America; 2 Center for Cell and Gene Therapy, Baylor College of Medicine, Houston, Texas, United States of America; University of Bergen, Norway

## Abstract

**Background:**

A large number of human tumor-associated antigens that are recognized by CD8^+^ T cells in a human leukocyte antigen class I (HLA-I)-restricted fashion have been identified. Special AT-rich sequence binding protein 1 (SATB1) is highly expressed in many types of human cancers as part of their neoplastic phenotype, and up-regulation of SATB1 expression is essential for tumor survival and metastasis, thus this protein may serve as a rational target for cancer vaccines.

**Methodology/Principal Findings:**

Twelve SATB1-derived peptides were predicted by an immuno-informatics approach based on the HLA-A*02 binding motif. These peptides were examined for their ability to induce peptide-specific T cell responses in peripheral blood mononuclear cells (PBMCs) obtained from HLA-A*02^+^ healthy donors and/or HLA-A*02^+^ cancer patients. The recognition of HLA-A*02^+^ SATB1-expressing cancer cells was also tested. Among the twelve SATB1-derived peptides, SATB1_565–574_ frequently induced peptide-specific T cell responses in PBMCs from both healthy donors and cancer patients. Importantly, SATB1_565–574_-specific T cells recognized and killed HLA-A*02^+^ SATB1^+^ cancer cells in an HLA-I-restricted manner.

**Conclusions/Significance:**

We have identified a novel HLA-A*02-restricted SATB1-derived peptide epitope recognized by CD8^+^ T cells, which, in turn, recognizes and kills HLA-A*02^+^ SATB1^+^ tumor cells. The SATB1-derived epitope identified may be used as a diagnostic marker as well as an immune target for development of cancer vaccines.

## Introduction

One of the most promising approaches in cancer therapy relies on harnessing the immune system to eradicate malignant cells [1], the success of which relies largely on the identification of suitable tumor-associated antigens (TAA) for generating effective cancer vaccines. It has been well-established that tumor cells express TAAs that can be recognized by CD8^+^ T cells in the context of human leukocyte antigen class I (HLA-I) molecules. A large number of TAAs and TAA-derived epitopes have been identified [2], [3], with some of these proteins and peptide derivatives already in clinical vaccine trials. Recent approvals of the immunotherapy-based vaccine/drug sipuleucel-T (Provenge) and ipilimumab (Yervoy) by the Food and Drug Administration (FDA) represent milestones in the field of cancer immunotherapy [4], [5]. And a phase III clinical trial of the gp100 peptide for melanoma also yielded highly encouraging results [6]. In addition, work from two independent groups underlined the importance of tumor-specific antigens in eliciting immune responses against a developing tumor [7], [8], undoubtedly further intensifying the efforts to search for novel tumor antigens for cancer immunotherapy.

Despite such promising results, success in cancer vaccine trials on the whole has been sporadic [9]–[11]. During the last couple of years, several TAAs that are expressed in different types of neoplasia have been identified [2], [3]. However, the majority of the antigens described thus far are dispensable for the survival and growth of the tumor cells, with the exception of a few TAAs such as telomerase [12], survivin [13] and anti-apoptotic members of the Bcl-2 family (Bcl-2, Bcl-X(L) and Mcl-2) [14]. Tumor cells may therefore have escaped surveillance by the immune system through loss and/or down-regulation of tumor antigens [15]. Consequently, targeting TAAs that are essential for survival and growth of tumor cells may better prevent immunoselection of antigen-loss variants as a result of vaccination and improve the efficacy of cancer immunotherapy [15], [16]. Such immunogenic tumor antigens that elicit minimal immune escape therefore represent the most optimal vaccine candidates for immunotherapy of cancer.

Special AT-rich sequence binding protein 1 (SATB1) is a nuclear factor that functions as a global chromatin organizer. It regulates gene expression by folding chromatin into loop domains, and tethering DNA domains to the SATB1 network structure [17]. SATB1 appeared to be over-expressed in aggressive breast cancer cell lines but absent or undetectable in normal and immortalized human mammary epithelial cells, suggesting a role of SATB1 in reprogramming chromatin organization and ultimately transcriptional profiles of breast tumors to promote growth and metastasis [18]. In addition, higher levels of SATB1 expression were associated with many other types of cancer, including laryngeal squamous cell carcinoma [19], endometrioid endometrial cancer [20], hepatocellular carcinoma [21], rectal cancer [22], cutaneous malignant melanoma [23], gastric cancer [24], [25], ovarian cancer [26], prostate cancer [27], lung cancer [28] and glioma [29]. Up-regulation of SATB1 in these types of cancers can promote tumor growth and metastasis. Since SATB1 is essential for tumor growth/survival and metastasis, immune escape by loss or down-regulation of SATB1 expression may impair sustained tumor cell growth and/or metastasis, thus making SATB1 an attractive target for anticancer vaccines against various types of cancers that express SATB1.

In this report, we describe the identification of SATB1-derived T cell epitopes for T cell recognition using an immuno-bioinformatics approach. We selected twelve peptides that were predicted to bind to the HLA-A*02 molecule. They were synthesized and evaluated *in vitro* for their ability to stimulate T cells in PBMCs from healthy subjects and/or cancer patients based on interferon-γ (IFN-γ) release. One of these peptides, SATB1_565–574_, was found to induce IFN-γ release in peripheral T cells from both healthy subjects and cancer patients. Importantly, SATB1_565–574_ -specific T cells were able to recognize and kill HLA-A*02^+^, SATB1-expressing tumor cells in an HLA-I-dependent manner. These results demonstrate the validity of the immuno-bioinformatics approach and suggest SATB1_565–574_ may represent a new tumor-specific epitope for cancer immunotherapy.

## Materials and Methods

### Healthy Donors and Cancer Patients

HLA-A*02^+^ prostate or ovarian cancer patients and ten HLA-A*02^+^ healthy subjects were enrolled in this study after written informed consent was obtained. All protocols were approved by the Institutional Review Board (IRB) at the Baylor College of Medicine prior to commencing studies. 20 mL of peripheral blood was obtained from each person, and peripheral blood mononuclear cells (PBMCs) were isolated by density gradient centrifugation using Lymphoprep (Nycomed Pharma AS; Oslo, Norway). Freshly isolated PBMCs were cryopreserved for later use in 1 mL freezing medium containing 90% FCS and 10% dimethyl sulfoxide (DMSO) at −140°C. HLA-A*02 expression in PBMCs obtained from cancer patients and healthy subjects was verified by flow cytometry with FITC-labeled HLA-A*02 mAb BB7.2 (BD Pharmingen; San Diego, CA, USA).

### Cell Lines

All breast cancer cell lines (MCF-7, CAMA-1, MDA-MB-134VI, MDA-MB-175VII, MDA-MB-361, DU4475, MDA-MB-231, MDA-MB-436, MDA-MB-453, MDA-MB-468), T2 cells (an HLA-A*02^+^ TAP-deficient cell line), prostate cancer cell lines (PC3, LNCaP and DU145), ovarian cancer cell line Ovcar-3 and lymphoma cell line Jeko-1 were purchased from American Type Culture Collection (ATCC; Manassas, VA, USA). An ovarian cancer cell line Skov-1 [30], [31] was a gift from Dr. Kunle Odunsi (Roswell Park Cancer Institute, NY, USA); a lymphoma cell line L1236 [32], [33] was a gift from Dr. Catherine M. Bollard (Baylor College of Medicine, Houston, USA). All cell lines were maintained in RPMI-1640 medium (Mediatech; Manassas, VA, USA), supplemented with 10% FBS, 1% L-glutamine, and 1% penicillin and streptomycin.

### Peptides

Twelve SATB1-derived peptides (Table 1) were predicted using BIMAS (http://www-bimas.cit.nih.gov/molbio/hla_bind/), SYFPEITHI (http://www.syfpeithi.de/), and Rankpep (http://bio.dfci.harvard.edu/Tools/rankpep.html) based on the HLA-A*02 binding motif. Epitopes that were predicted by at least two of these algorithms were selected for further testing. The peptides were synthesized by a solid-phase method using a peptide synthesizer (AApptec, Inc.; Louisville, KY, USA), purified by reverse-phase high-performance liquid chromatography and validated by mass spectrometry. The synthesized peptides were dissolved in DMSO at a concentration of 10 mg/mL and stored at −80°C until further use. One peptide (SATB1_544–552_) was excluded from the study due to the difficulty of peptide synthesis.

**Table 1:** A list of predicted HLA-A*02 binding peptides derived from SATB1.

				Binding score		
SATB1 peptides	HLA restriction	Position	Sequence	BIMAS^a^	SYFPEITHI^b^	Rankpep^c^
SATB1_105–113_	HLA-A2	105113	MLFNQLIEM	71.872	21	87
SATB1_658–666_	HLA-A2	658–666	ILQSFIQDV	1033.404	27	86
SATB1_544–552_	HLA-A2	544–552	TLWENLSMI	816.565	25	95
SATB1_156–164_	HLA-A2	156–164	MLQDVYHVV	298.138	22	88
SATB1_514–522_	HLA-A2	514–522	ALFAKVAAT	63.417	25	80
SATB1_325–333_	HLA-A2	325–333	QLLNQQYAV	257.342	24	81
SATB1_315–323_	HLA-A2	315–323	QLVNQQLVM	2.037	15	73
SATB1_72–80_	HLA-A2	72–80	TMLPVFCVV	144.784	22	69
SATB1_623–631_	HLA-A2	623–631	RLPPRQPTV	69.552	25	54
SATB1_565–574_	HLA-A2	565–574	AIYEQESNAV	125.472	25	11.261
SATB1_196–205_	HLA-A2	196–205	LLKDMNQSSL	5.211	24	10.011
SATB1_214–223_	HLA-A2	214–223	SMISSIVNST	12.379	23	7.812

Note: ^a^ Predicted T(1/2) of disassociation in minutes;

b and ^c^ Higher values represent better binders.

### *In vitro* Stimulation of Peptide-specific T Cells in PBMCs

PBMCs (1×10^5^ cells/well) from either healthy subjects or cancer patients were incubated with standard peptide concentrations of 20 µg/mL per peptide [34]–[37] in 96-well U-bottom microplates (BD; Franklin Lakes, NJ, USA) in 200 µL of T-cell medium (TCM), consisting of RPMI 1640 (Mediatech; Manassas, VA, USA), 10% human AB serum (Valley Biomedical, Winchester, USA), 50 µM of 2-mercaptoethanol, 100 IU/mL of interleukin-2 (IL-2), and 0.1 mM MEM nonessential amino acid solution (Invitrogen; grand island, NY, USA). Half of the TCM was removed and replaced with fresh TCM containing peptides (20 µg/mL) every 5 days. After 14 days of culture, the cells were harvested and tested for their ability to produce IFN-γ in response to T2 cells (1×10^4^ cells/well), which were pre-loaded with either SATB1 peptide (5 µg/mL) or a control peptide (an irrelevant HLA-A*02 binding EBV peptide: GLCTLVAML ) as a negative control. After 18 hours of incubation, supernatants were collected, and IFN- γ release was determined by ELISA assay.

### Rapid Expansion Protocol (REP) for SATB1 Peptide-specific T Cells

SATB1 peptide-specific T cells were expanded by a rapid expansion protocol (REP) as previously described [38] with a slight modification. Briefly, on day 0, 0.1–0.5×10^6^ SATB1 peptide-specific T cells were cultured in a T25 flask with 20 mL RPMI-1640 supplemented with 10% human AB serum, 50 µM of 2-mercaptoethanol, 30 ng/mL OKT3 antibody (Ortho Biotech; Bridgewater, NJ, USA) and 30 ng/mL anti-CD28 antibody (R&D Systems; Minneapolis, MN, USA), together with 20×10^6^ irradiated allogeneic PBMCs and 5×10^6^ irradiated Epstein Barr Virus (EBV) transformed B cells as feeder cells. Flasks were incubated upright at 37°C in 5% CO_2_. IL-2 (300 IU/mL) was added on day 1, and on day 5, half of the cell culture supernatant was removed and replenished with fresh medium containing 300 IU/mL IL-2. 14 days after initiation of the REP, cells were harvested and cryopreserved for future experiments.

### Depletion of CD4^+^ or CD8^+^ T Cells from PBMCs

CD4^+^ T cells or CD8^+^ T cells were positively depleted from PBMCs according to the manufacturer’s instruction using monoclonal anti-CD4-coated or monoclonal anti-CD8-coated Dynabeads (Dynal Biotech ASA; Oslo, Norway). PBMCs depleted of CD4^+^ T or CD8^+^ T cells were verified by flow cytometry.

### ELISA Assay

Cytokine release was measured by coating 96-well ELISA plates (Thermo Fisher Scientific; Rochester, NY, USA) with 1 µg/mL anti-human IFN-γ (Pierce Biotechnology; Rockford, IL, USA) overnight at 4°C. The plate was washed six times with PBS containing 0.05% Tween-20 (wash solution) to remove unbound coating antibody, and blocked with 1% BSA/PBS at room temperature for 2 hrs. Afterwards, 50 µL supernatant was added to each well and incubated at room temperature for 1 hr, then 50 µL of 0.5 µg/mL biotinylated anti-human IFN-γ (Pierce Biotechnology; Rockford, IL, USA) was added and plates were incubated for an additional 1 hr at room temperature. After incubation, plates were washed and incubated for 30 min with Poly-HRP-Streptavidin (Thermo Fisher Scientific; Rochester, NY, USA) diluted 1∶5000 in PBS/1% BSA. Plates were washed and 100 µL of TMB substrate solution (Sigma-Aldrich Co.; St. Louis, MO, USA) was added per well. The colorimetric reaction was stopped using 2N H_2_SO4 and plates were read at 450 nm using an ELISA plate reader.

### RNA Extraction and RT-PCR

RNA extraction and RT-PCR was carried out as reported previously [39]. In brief, total RNA was extracted from cancer cells with 1 mL Trizol reagent (Invitrogen; Carlsbad, CA, USA). Three micrograms of RNA was reverse-transcribed to cDNA in 30 µl volume and 1 µl of each cDNA was used in subsequent PCR reaction with a pair of SATB1 specific primers: Primer 1∶5′-TGCAAAGGTTGCAGCAACCAAAAGC-3′; 5′-AACATGGATAATGTGGGGCGGCCT-3′. GAPDH was used as loading control: primer 1∶5′-TGATGACATCAAGAAGGTGGTGAAG-3′; Primer 2∶5′-TCCTTGGAGGCCATGTGGGCCAT-3′. The PCR reaction was carried out under the following conditions: 95°C for 1 min, 95°C for 40 s, 60°C for 30 s, 72°C for 40 s, total 40 cycles, 72°C for 5 min, and GAPDH was run for 25 cycles. Equal amounts of PCR products were then loaded and detected by gel electrophoresis.

### Western Blot

Whole cell extracts were prepared and resolved in SDS-PAGE gels. The proteins were transferred to PVDF membrane (Bio-Rad Laboratories, Inc., Hercules, CA, USA) and further incubated with the SATB1 antibody (BD Biosciences, San Jose, CA, USA). LumiGlo Chemiluminescent Substrate System from KPL (Gaithersburg, MD, USA) was used for protein detection.

### FACS Analysis

Cells (0.5×10^6^) were stained with either FITC-anti-CD8, PE-Cy5-anti-CD4 **(**Both from eBioscience, San Diego, CA, USA) or FITC-anti-HLA-A*02 (BD Pharmingen; San Diego, CA, USA) in PBS containing 2% FBS on ice for 30 min, and then washed twice in PBS. Afterwards, cells were re-suspended in 500 µl PBS and analyzed using a FACScalibur machine. For DimerX HLA-A*02:Ig staining, SATB1 reactive CD8^+^ T cells were incubated with purified HLA-A*02:Ig dimer (BD Biosciences, San Jose, CA, USA) loaded with a given peptide, and then stained with FITC anti-mouse IgG1 (BD Biosciences, San Jose, CA, USA). Flow cytometry was performed on a FACScalibur machine.

### Cytotoxicity Assay

SATB1-derived peptide-specific T cells were tested for cytotoxicity against peptide-loaded T2 cells, two HLA-A*02^+^ SATB1^+^ cancer cell lines (Skov-1 and Jeko-1) and an HLA-A*02 negative SATB1^+^ PC3 cell line as a negative control by a lactate dehydrogenase (LDH) assay (Promega; Madison, WI, USA). The assay was performed in accordance with the manufacturer’s instructions. LDH release was calculated based on the following formula:

Cytotoxicity (%) = (Experimental – Effector Spontaneous – Target Spontaneous LDH release)/(Target Maximum – Target Spontaneous LDH release)×100.

Spontaneous release was determined by using the supernatant of the target cells alone or effector cells alone, and the maximum release was determined by using the supernatant of target cells incubated with a lysis solution included in LDH kit.

### Statistics

Student’s t-test was used to analyze quantitative differences between the experimental wells and controls in ELISA assays. P<0.05 was considered significant.

## Results

### SATB1 mRNA is Expressed in a Variety of Cancers

**Figure 1:**
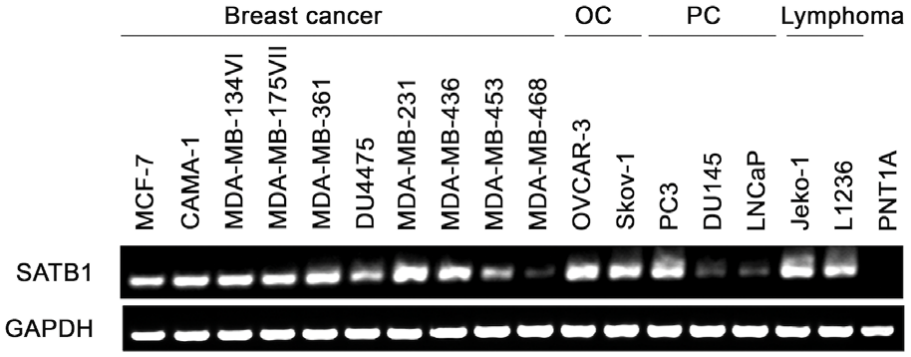
SATB1 mRNA was highly expressed in various types of cancers. The mRNA expression for SATB1 in different cell lines was determined by RT-PCR. The breast cancer cell lines (MCF-7, CAMA-1, MDA-MB-134VI, MDA-MB-175VII, MDA-MB-361, DU4475, MDA-MB-231, MDA-MB-436, MDA-MB-453 and MDA-MB-468), prostate cancer (PC) cell lines (PC3, LNCaP and DU145), ovarian cancer (OC) cell lines (Ovcar-3 and Skov-1), lymphoma cell lines (Jeko-1 and L1236) and a normal prostate epithelial cell line PNT1A were included as a control. Results are representative of three independent experiments.

To examine whether SATB1 is expressed in cancer cells, mRNA expression for SATB1 in various types of tumor cells was performed by RT-PCR. As shown in Figure 1, SATB1 mRNA was highly expressed in a variety of cancers including breast cancer, ovarian cancer, prostate cancer as well as lymphoma. While the normal prostate epithelial cell line, PNT1A did not express SATB1 mRNA.

### Induction of SATB1-derived Peptide-specific CTLs in Healthy Donors

First, we obtained PBMCs from 10 HLA-A*02^+^ healthy donors to determine whether SATB1-reactive T cell precursors were present in these healthy subjects. The cells were stimulated *in vitro* for two weeks with each of the SATB1-derived peptides containing HLA-A*02-binding motif (Table 1). At the end of peptide stimulation, supernatants from the cultures were analyzed by ELISA to detect IFN-γ release in response to T2 cells pulsed with or without corresponding peptides. As shown in Table 2, nearly all of the 11 SATB1-derived peptides (10/11) were capable of inducing peptide-specific T cell responses in at least one of the healthy subjects. In particular, the peptide SATB1_565–574_ induced higher level of IFN-γ release (>900 pg/mL) in 4 out of 10 healthy subjects, indicating its high immunogenicity and potential in expanding antigen-specific T cells in healthy subjects.

**Table 2:** Recognition of the peptides by in vitro-stimulated T cells from the PBMCs of ten HLA-A*02^+^ healthy subjects.

	# 1	# 2	# 3	# 4	# 5	# 6	# 7	# 8	# 9	# 10
SATB1_105–113_	0	0	467	0	138	0	0	677	0	0
SATB1_658–666_	0	0	0	0	127	0	0	0	106	0
SATB1_156–164_	582	152	109	109	0	0	0	0	465	168
SATB1_514–522_	172	0	259	0	0	0	0	0	203	0
SATB1_325–333_	366	0	823	147	112	0	0	729	111	0
SATB1_315–323_	0	0	0	0	0	0	0	0	0	0
SATB1_72–80_	208	0	0	171	0	0	0	742	132	214
SATB1_623–631_	0	0	0	93	316	116	0	0	0	301
**SATB1_565_**_–**574**_	**1106**	**0**	**0**	**906**	**954**	**90**	**0**	**1032**	**0**	**99**
SATB1_196–205_	352	0	0	0	0	0	0	0	0	0
SATB1_214–223_	0	0	0	0	0	0	0	1061	0	0

**Note:** Values denote concentrations of IFN-γ (pg/ml) in the supernatants of T cells stimulated with SATB1 peptide-loaded T2 cells minus that of T cells stimulated with unloaded T2 cells.

### Presence of SATB1-derived Peptide Specific CTLs in Cancer Patients

Our analysis indicated that peptide-specific T cells against SATB1_565–574_ were found in ∼60% of healthy subjects, we therefore reasoned that CTL precursors that could recognize this peptide might also be abundant in PBMCs from cancer patients. To test our hypothesis, we examined whether peptide SATB1_565–574_ could induce peptide-specific CTLs from PBMCs of HLA-A*02^+^ ovarian cancer patients. PBMCs from three HLA-A*02^+^ ovarian cancer patients were collected and stimulated *in vitro* with peptide SATB1_565–574_. As shown in Table 3, peptide SATB1_565–574_ was able to induce peptide-specific CTLs from PBMCs of ovarian cancer patients, indicating that the peptide is highly immunogenic not only in healthy subjects but also in cancer patients. We also determined whether this peptide candidate could induce peptide-specific CTLs from PBMCs of HLA-A*02^+^ prostate cancer patients. As was the case with ovarian cancer patients, peptide SATB1_565–574_ similarly induced peptide-specific CTLs from PBMCs of 5 prostate cancer patients as well (Table 3), indicating CTL precursors that recognize this peptide are abundant in PBMCs from cancer patients.

**Table 3:** Recognition of the peptide by in vitro-stimulated T cells from the PBMCs of HLA-A*02^+^ cancer patients.

	Ovarian cancer patients			Prostate cancer patients				
	MD	CH	EL13	#1	#2	#3	#4	#5
SATB1_565–574_	1000	16	231	1813	1946	873	1194	242

**Note:** Values denote concentrations of IFN-γ (pg/ml) in the supernatants of T cells stimulated with SATB1 peptide-loaded T2 cells minus that of T cells stimulated with unloaded T2 cells.

### SATB1-derived Peptide Induced CD8^+^ T Cell-dependent Responses

**Figure 2:**
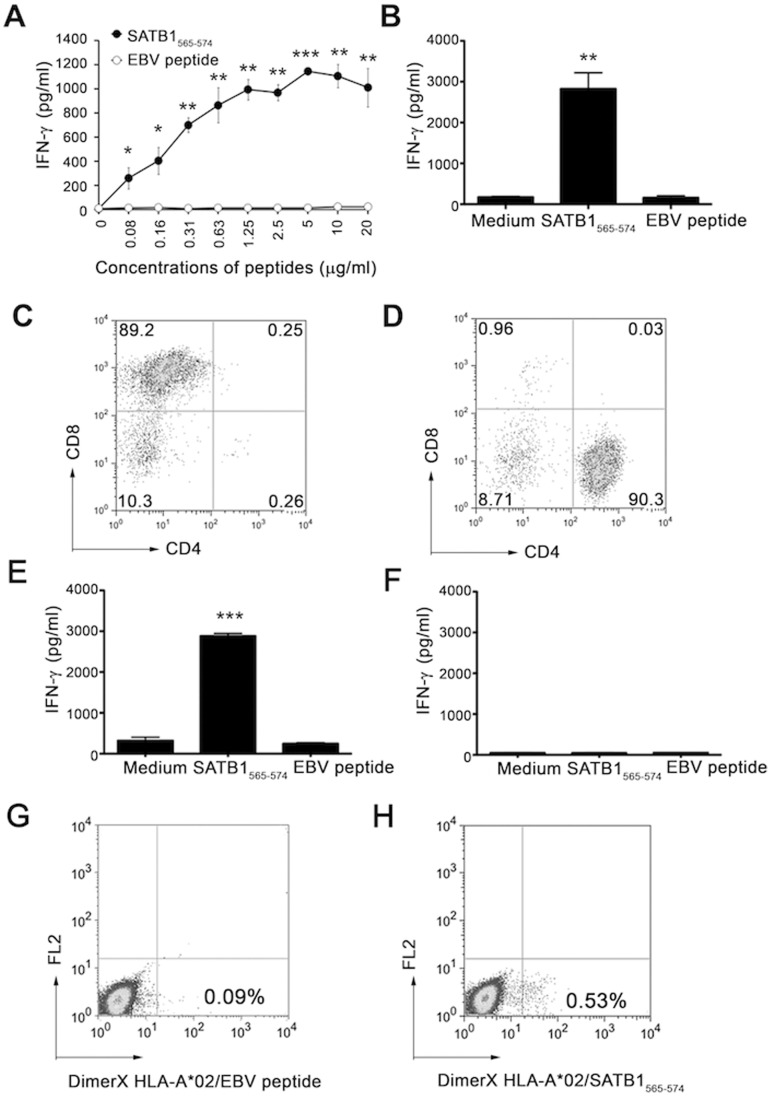
SATB1-derived peptide SATB1_565–574_ **induced CD8^+^ T cell-dependent responses.** The recognition of T2 cells pre-loaded with titrated concentrations of peptides (0–20 µg/ml) by expanded SATB1_565–574_-specific T cells from healthy donor #1 was tested by ELISA assay (A). The expanded SATB1-reactive PBMCs (B) were co-incubated with T2 cells (1×10^4^ cells/well) alone in complete medium (CM), or with T2 cells pre-loaded with either SATB1_565–574_ (5 µg/mL) or a control EBV peptide as a negative control. Cells were incubated for 18–24 hours, the IFN-γ secretion in the supernatant was determined by ELISA assay. SATB1-reactive PBMCs depleted of CD4^+^ T cells (C) and SATB1-reactive PBMCs depleted of CD8^+^ T cells (D) were verified by flow cytometry. The recognitions of T2 cells pulsed with SATB1_565–574_ by SATB1-reactive PBMCs depleted of either CD4^+^ T cells (E) or CD8^+^ T cells (F) were determined by ELISA. SATB1 reactive CD8^+^ T cells were incubated with purified HLA-A2:Ig dimer loaded with a control EBV peptide (G) or with peptide SATB1_565–574_ (H), and then stained with FITC anti-mouse IgG1. Cells were analyzed using a FACScalibur machine. Data (from A, B, E and F) are plotted as means ± SD. Results are representative of at least three independent experiments. *P<0.05, **P<0.01, ***P<0.001 versus controls (T2 cells alone or T2 cells pulsed with a control EBV peptide).

To further analyze SATB1_565–574_ peptide-specific T cells, we next expanded SATB1_565–574_ peptide-specific T cells identified in Tables 2 and 3 in order to obtain a sufficient number of these cells. To this end, we performed peptide titration experiments to determine the optimal peptide concentration for loading T2 cells for T cell recognition. As shown in Figure 2A, T2 cells could be sensitized by peptide SATB1_565–574_ but not a control EBV peptide for T cell recognition at a concentration of 0.08 µg/mL, and the binding sites of HLA-A*02 molecules on T2 cells became saturated at 5 µg/mL. Further increasing the concentrations of SATB1_565–574_ failed to enhance production of IFN-γ. Therefore, we consistently used the peptide concentration of 5 µg/mL for pre-loading T2 cells in our ELISA assays. The expanded T cells maintained antigen-specificity and secreted significant amounts of IFN-γ after stimulation with T2 cells pulsed with the corresponding peptides, but not with a control EBV peptide (Figures 2A and B). To obtain direct evidence on the subsets of the responding T cells depicted in Figure 2B, the expanded SATB1 peptide-reactive PBMCs were depleted for either CD4^+^ T cells (Figure 2C) or CD8^+^ T cells (Figure 2D) prior to incubation with peptides for ELISA assays. As shown in Figure 2E, CD8^+^ T cells, not CD4^+^ T cells (Figure 2F), obtained from expanded PBMCs responded to SATB1_565–574_ pulsed T2 cells, indicating that the T cell response induced by SATB1_565–574_ was dependent on CD8^+^ T cells. Furthermore, dimerX HLA-A*02:Ig staining (Figure 2G and H) and IFN-γ intracellular staining (Figure S1) also demonstrated that SATB1_565–574_ induced peptide specific, HLA-A*02 restricted CD8^+^ T cell-dependent responses.

### Recognition and Killing of Cancer Cells by the SATB1-derived Peptide-specific CD8^+^ T Cells in an HLA-I Restricted Manner

**Figure 3:**
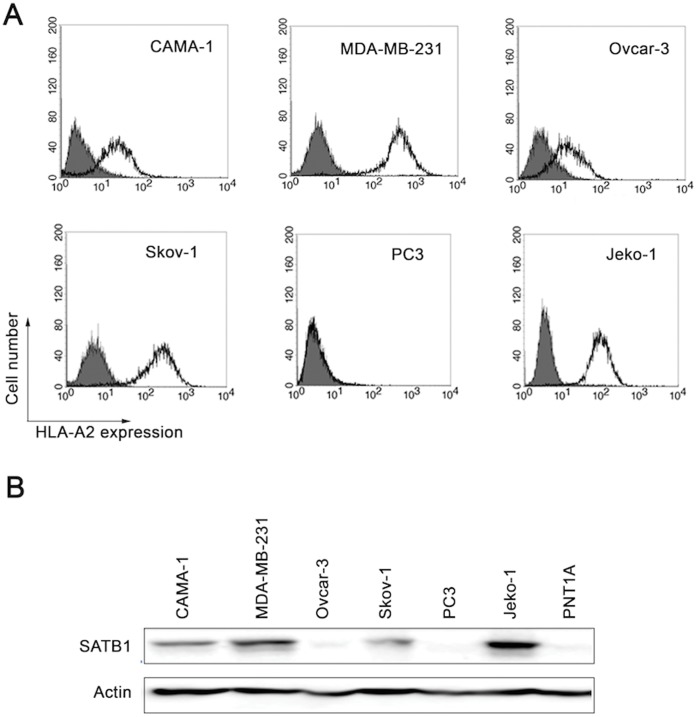
HLA-A*02 and SATB1 protein expression in tumor cell lines. (A) Cells were stained with FITC-anti-HLA-A*02 in PBS containing 2% FBS. Then, cells were washed and re-suspended in PBS and analyzed using a FACScalibur machine. (B) The expression of SATB1 protein in various tumor cells was determined by western blot. Actin was used as a loading control.

**Figure 4:**
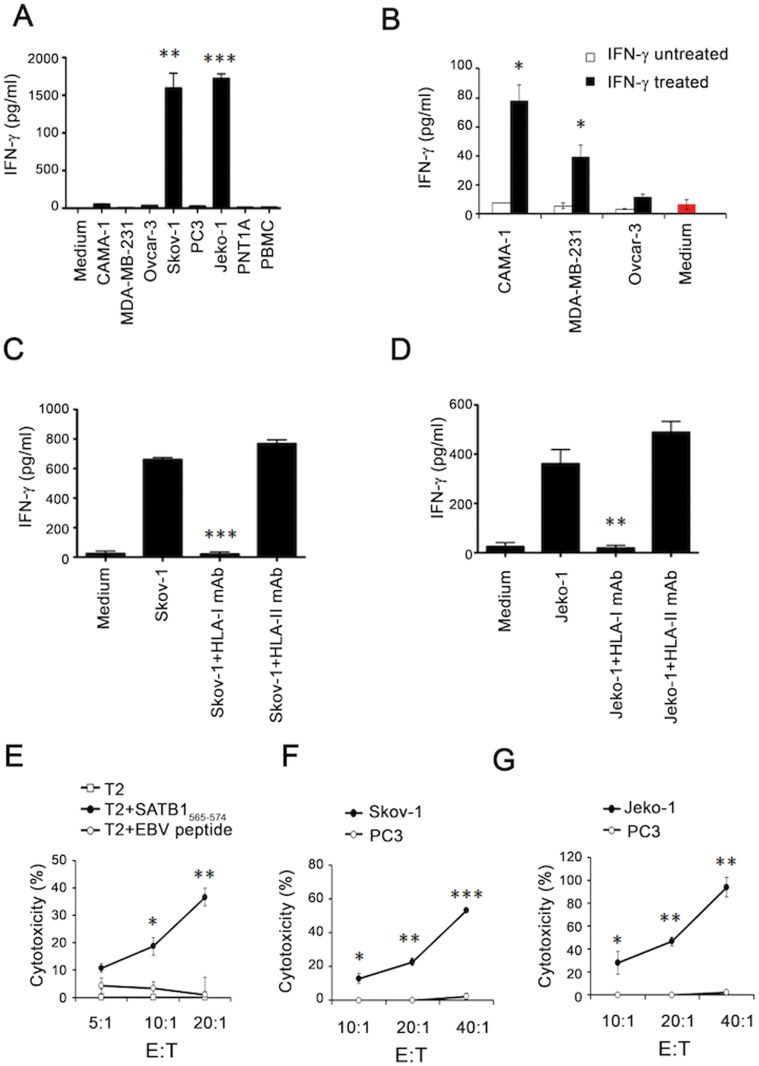
SATB1_565–574_-specific CD8^+^ T cells recognized and killed HLA-A*02^+^ SATB1-expressing cancer cells in a HLA-I dependent manner. (A) SATB1_565–574_-specific T cells from healthy donor #1 were cultured alone in medium or co-incubated with various types of tumor cells as well as normal cells (PNT1A and PBMC); (B) Tumor cells were pretreated without or with IFN-γ (10 ng/mL) for 48 hours before incubation with SATB1_565–574_-specific T cells. SATB1_565–574_-specific T cells were cultured in medium alone as a negative control (red column); To block HLA-dependent responses, either anti-HLA-I mAb (W6/32) or HLA-II mAb was added into cell cultures during incubation of SATB1_565–574_-specific T cells with Skov-1 (C) or Jeko-1 cells (D). Cells were incubated for 18 –24 hours, the IFN-γ secretion in the supernatant was determined by ELISA assay. SATB1_565–574_-specific CD8^+^ T cells were tested for cytotoxicity against T2 cells pulsed with or without peptides (E), Skov-1 (F) and Jeko-1 (G) by the LDH assay. HLA-A*02 negative PC3 cells were used as a negative control in the LDH assay. Data from A–G are plotted as means ± SD. Results are representative of three independent experiments. *P<0.05, **P<0.01, ***P<0.001 versus controls.

Based on our results thus far, SATB1_565–574_ specific CD8^+^ T cells were used in subsequent experiments. To determine whether SATB1-derived peptide-specific T cells were able to recognize and kill HLA-A*02^+^, SATB1-expressing cancer cells, we used an HLA-A*02^−^ SATB1 mRNA positive prostate cancer cell line PC3 (as a negative control) and five HLA-A*02^+^ SATB1 mRNA positive cancer cell lines. The expression of SATB1 mRNA in these 6 cell lines was previously examined by RT-PCR (Figure 1) and HLA-A*02 expression was verified by flow cytometry (Figure 3A). In addition, we also checked the expression of SATB1 protein among these cell lines (Figure 3B), and found that all cancer cell lines expressed SATB1 protein except PC3 and Ovcar-3. Therefore, Ovcar-3 was also regarded as a negative control. As shown in Figure 4A, SATB1_565–574_ -specific T cells could recognize HLA-A*02^+^ SATB1 expressing Skov-1 and Jeko-1 cells, but not PC3 or Ovcar-3 cells. The other two HLA-A*02^+^ SATB1 expressing cancer cell lines (CAMA-1, MDA-MB-231) could only be recognized when they were pre-treated with IFN-γ (Figure 4B), suggesting that either enhanced HLA-A*02 expression on the cell surfaces (Figure S2) or induction of immunoproteasomes by IFN-γ, may facilitate the presentation of the correct epitope to cell surfaces for T cell scrutiny. In contrast, normal cells, including PNT1A and autologous PBMCs, could not be recognized by SATB1_565–574_ -specific T cells (Figure 4A). In addition, SATB1_565–574_-specific T cells did not recognize in vitro-differentiated Th subsets, including Th1, Th2 and Th17 cells (Figure S3).

To determine whether the recognition of cancer cells by SATB1_565–574_-specific CD8^+^ T cells was HLA-I restricted, we co-cultured SATB1_565–574_ -specific T cells with either Skov-1 or Jeko-1 cells in the presence of either anti-HLA-I mAb (W6/32) or anti-HLA-II mAb. As shown in Figure 4C and D, T cell responses were completely inhibited by the addition of anti-HLA-I mAb, but not anti-HLA-II (HLA-DP mAb), which suggests that the recognition of tumor cells by SATB1_565–574_-specific CD8^+^ T cells is HLA-I restricted.

To further examine whether SATB1_565–574_-specific CD8^+^ T cells were able to kill HLA-A*02^+^, SATB1-expressing cancer cells, we performed cytotoxicity assays. As shown in Figure 4E, SATB1_565–574_-specific T cells killed T2 cells pulsed with SATB1_565–574,_ but not T2 cells alone or those pulsed with a control EBV peptide. Importantly, SATB1_565–574_-specific T cells were able to kill HLA-A*02^+^, SATB1 expressing Skov-1 and Jeko-1 cells, but not HLA-A*02^−^ PC3 cells (Figure 4F and G). These results suggest that SATB1_565–574_ -specific T cells recognize a T cell epitope that is endogenously processed and presented by tumor cells.

## Discussion

It is well-established that CD8^+^ T cells play a critical role in controlling tumor development and progression. Peptide epitopes derived from TAAs can be recognized as antigens by T cells in the context of MHC-I molecules [40], [41]. Identification of ideal TAAs and their peptides that are recognized by T cells are essential for the development of effective cancer vaccines. Ideal TAAs such as anti-apoptotic proteins [14], telomerase [12] and survivin [13], being mandatory for tumor growth/survival, may represent optimal targets for vaccine mediated immunotherapy of cancer. SATB1 is highly expressed in many types of human cancers and up-regulation of SATB1 expression is essential for tumor survival and metastasis, thus SATB1 represents one of such ideal TAAs.

The aim of the current study was to identify HLA-A*02 binding SATB1-derived epitopes recognized by CD8^+^ T cells in PBMCs of healthy subjects and cancer patients. Twelve SATB1-derived peptides were predicted using BIMAS, SYFPEITHI, and Rankpep based on the HLA-A*02 binding motif. Peptides that were predicted by at least 2 of 3 programs were selected for further testing for their ability to stimulate PBMCs from healthy subjects and/or cancer patients. Further studies showed that 10 out of 11 synthesized SATB1-derived peptides were capable of inducing peptide-specific T cell responses in at least one out of 10 healthy subjects. Specifically, SATB1_565–574_ was found to induce higher level of IFN-γ release (>900 pg/mL) in 4 out of 10 healthy subjects, indicating that this peptide may be immunogenic and potentially capable of expanding antigen-specific T cells in healthy subjects. Furthermore, SATB1_565–574_ induced frequently specific T cell responses in PBMCs of ovarian cancer patients as well as in PBMCs from prostate cancer patients. Importantly, SATB1_565–574_ peptide-specific T cells recognized and killed HLA-A*02^+^ SATB1-expressing Jeko-1, Skov-1 cells as well as IFN-γ-treated breast cancer cell lines (CAMA-1 and MDA-MB-231), suggesting this peptide is naturally processed by cancer cells.

SATB1 is a global chromatin organizer and transcription factor and has emerged as a key factor integrating higher-order chromatin architecture with gene regulation [42]. SATB1 promotes tumor growth and metastasis by reprogramming chromatin organization and transcription profiles, and is highly expressed in a variety of cancers including breast cancer [18], laryngeal squamous cell carcinoma [19], endometrioid endometrial cancer [20], hepatocellular carcinoma [21], rectal cancer [22], cutaneous malignant melanoma [23], gastric cancer [24], [25], ovarian cancer [26], prostate cancer [27], lung cancer [28] and glioma [29]. In tumor cells with high levels of SATB1, genes that promote tumor progression and metastasis were up-regulated, whereas genes that inhibit tumor metastasis were repressed [17]; while silencing of SATB1 greatly reduced the invasive and metastatic capacity of cancer cells [43]. Thus SATB1 is required and essential for tumor growth/survival and metastasis, and down-regulation or loss of SATB1 expression may impair sustained tumor growth and metastasis. These characteristics make SATB1 an attractive target for development of effective cancer vaccines against various types of cancer. Here, we have described an HLA-A*02 binding SATB1-derived epitope, SATB1_565–574_. This is, to our knowledge, the first report to identify and characterize a SATB1-derived epitope that is recognized by CD8^+^ T cells.

Although SATB1_565–574_ peptide-specific T cells recognized and killed HLA-A*02^+^ SATB1-expressing Jeko-1 and Skov-1 cells, however, they did not directly recognize the other two HLA-A*02^+^ SATB1^+^ breast cancer cells tested in this study including CAMA-1 and MDA-MB-231. These differences may arise from the different cell types harboring different sets of proteasomes [44], thus leading to different repertoire of peptides associated with MHC-I in different cell types. Indeed, when CAMA-1 and MDA-MB-231 were pretreated with an inducer of immunoproteasomes-IFN-γ, they could then be recognized by SATB1_565–574_ peptide-specific T cells (Figure 4B), suggesting that IFN-γ may enhance induction of immunoproteasomes, thus leading to the correct presentation of SATB1_565–574_ to the tumor cell surface for T cell scrutiny. The enhanced expression of HLA-A*02 on cell surface by IFN-γ (Figure S2) is another possibility that make treated tumor cells recognized by SATB1_565–574_ peptide-specific T cells.

It should be noted that SATB1 is not only highly expressed in various types of malignant tumor, its expression was also detectable in thymocytes and progenitor cells including osteoblasts, the basal layer of the epidermis, amyloblasts, and embryonic stem cells [45]–[47]. These cells may be targeted by vaccine-induced SATB1-peptide specific CD8^+^ T cells. However, the fact that SATB1_565–574-_specific T cell responses are easily induced in healthy donors as well as cancer patients, suggests that a high frequency of SATB1-specific CD8^+^ T cell precursors is present in the circulation, which indicates that anti-cancer vaccines targeting SATB1 may be safe and may not induce autoimmunity. Indeed, SATB1_565–574_ peptide-specific T cells did not recognize autologous PBMC in vitro (Figure 4A). Furthermore, although SATB1 plays an important role during T cell differentiation, SATB1_565–574_ -specific T cells did not recognize in vitro-differentiated Th cell subsets, including Th1, Th2 and Th17 cells (Figure S3). Besides, the peptide SATB1_565–574_ -HLA-I complexes on the surface of normal cells may be too low to reach the threshold for T cell scrutiny; while in cancer cells, over-expression of SATB1 results in increased presentation of the peptide on the cell surface for T cell recognition. Thus, the difference in SATB1 expression on normal and malignant cells may explain the reason that SATB1-specific T cells in healthy donors do not cause autoimmunity, SATB1-targeted immunotherapy is expected to be safe to treat cancer patients. Nevertheless, the risk of autoimmunity induced by SATB1-targeted immunotherapy should be still considered in future clinic trials and impact of SATB1-specific T cells on developing T cells in vivo needs further exploration.

The FDA has recently approved a cancer vaccine, Sipuleucel-T, for the treatment of patients with advanced prostate cancer based on a phase III study [4]. Sipuleucel-T is prepared from autologous PBMCs containing antigen presenting cells that are incubated with a recombinant protein composed of a PAP linked to granulocyte-macrophage colony-stimulating factor (GM-CSF). Sipuleucel-T presumably works in part by augmenting PAP-specific CD8^+^ T cell responses, further demonstrating the importance of tumor antigen-specific CD8^+^ T cells induced by cancer vaccines. So far, Sipuleucel-T is the first cellular immunotherapeutic agent approved by the FDA to be used for the treatment of cancer patients. The FDA approval of Sipuleucel-T as a therapeutic cancer vaccine not only validates the efficacy of cancer immunotherapy, but also provides a strong impetus in the field of cancer immunology [1]. Therefore, identification and development of more novel TAAs including SATB1 and peptide derivatives recognized by CTLs is definitely essential to facilitate the development of effective cancer vaccines. Although, a large number of human TAAs have been identified in melanoma and other types of cancer [2], much less is known about ovarian and lymphoma tumor antigens, thus impeding the development of cancer immunotherapy for patients with ovarian cancer and lymphoma. In this study, we find that SATB1 is also highly expressed in ovarian cancer cells and lymphoma cells, and importantly SATB1-specific T cells can recognize and kill these cancer cells, suggesting that identification of SATB1 as a tumor antigen has important implications for the development of potentially therapeutic vaccines against ovarian cancer, lymphoma and other types of cancer as well.

In summary, we have identified a novel SATB1-derived CD8^+^ T cell epitope SATB1_565–574_. Since SATB1 expression is strongly up-regulated in various types of cancer, SATB1_565–574_ may serve as a diagnostic tool or an immunotherapeutic target of cancer vaccines for ovarian cancer, lymphoma and other types of cancer.

## Supporting Information

Figure S1 SATB1_565–574_ induced peptide-specific CD8^+^ T cell-dependent responses. SATB1_565–574_- reactive T cells were co-cultured with T2 cells loaded with EBV peptide as a negative control (A) or peptide SATB1_565–574_ (B) in the presence of GolgiStop in a 48-well plate for 4 hrs at 37°C. Cells were then stained with FITC conjugated anti-CD8 and PE conjugated anti-IFN-γ, and analyzed on a FACScalibur machine.
(TIFF)

Figure S2 IFN-γ enhanced expression of HLA-A*02 molecules on surfaces of tumor cells. Cells were pre-treated without (Green line) or with (red line) IFN-γ at 10 ng/mL for 48 hours, and then were stained with FITC-anti-HLA-A*02. Afterwards, cells were re-suspended in 500 µl PBS and analyzed using a FACScalibur machine. The grey histograms represent isotype controls.
(TIFF)

Figure S3 SATB1_565–574_ -specific T cells were not able to recognize in vitro-differentiated Th cell subsets. Different T cell subsets (Th1, Th2, Th17 and PBMC) were co-incubated without or with SATB1_565–574_ -specific CD8^+^ T cells (0.1×10^6^) in 96-well plate, respectively. Cells were incubated for 18–24 hours, the IFN-γ secretion in the supernatant was determined by ELISA assay. T2 cells loaded with SATB1_565–574_ were used as positive control. ***P<0.001 versus control (SATB1_565–574_–specific T cells stimulated with unloaded T2 cells).
(TIFF)

File S1 Supplemental Materials and Methods.
(DOCX)
