# Supplementary figures and images for: Identification of Special AT-Rich Sequence Binding Protein 1 as a Novel Tumor Antigen Recognized by CD8+ T Cells: Implication for Cancer Immunotherapy
Source: PLoS One. 2013 Feb 21;8(2):e56730. doi: 10.1371/journal.pone.0056730 (PMC3578933; doi:10.1371/journal.pone.0056730)

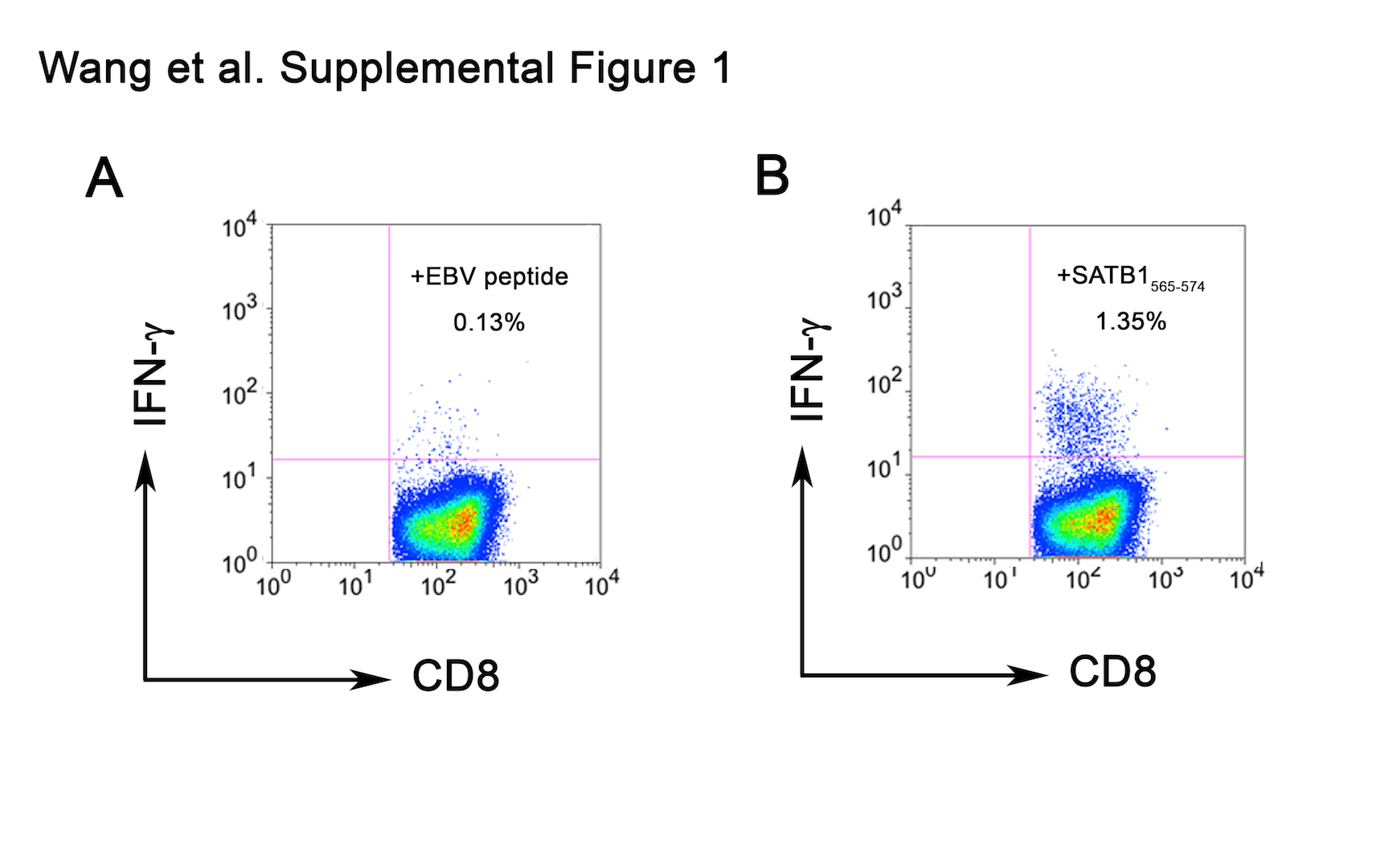

Supplement: Figure S1 — SATB1565–574 induced peptide-specific CD8+ T cell-dependent responses. SATB1565–574- reactive T cells were co-cultured with T2 cells loaded with EBV peptide as a negative control (A) or peptide SATB1565–574 (B) in the presence of GolgiStop in a 48-well plate for 4 hrs at 37°C. Cells were then stained with FITC conjugated anti-CD8 and PE conjugated anti-IFN-γ, and analyzed on a FACScalibur machine. (TIFF) [file pone.0056730.s001.tiff]

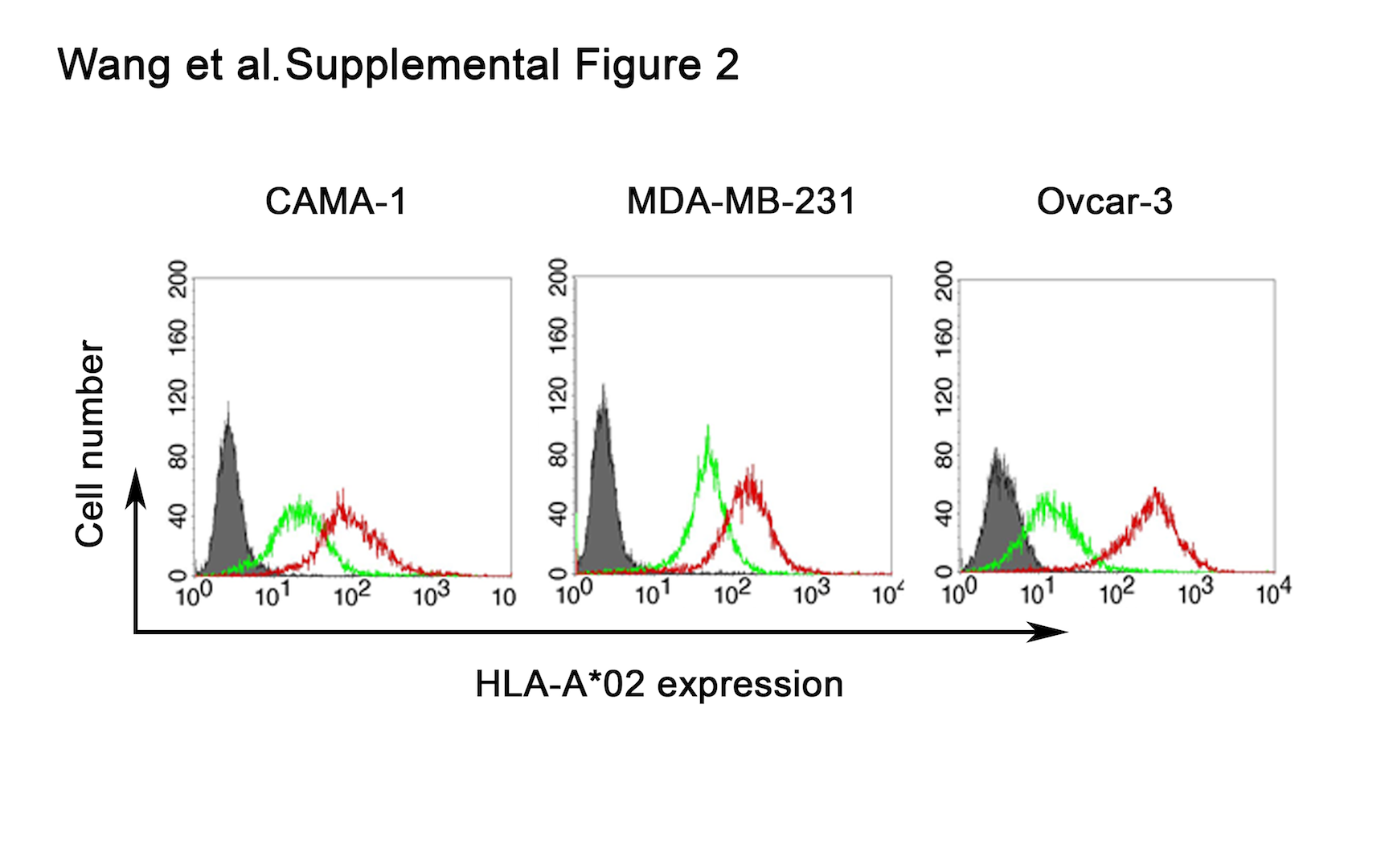

Supplement: Figure S2 — IFN-γ enhanced expression of HLA-A*02 molecules on surfaces of tumor cells. Cells were pre-treated without (Green line) or with (red line) IFN-γ at 10 ng/mL for 48 hours, and then were stained with FITC-anti-HLA-A*02. Afterwards, cells were re-suspended in 500 µl PBS and analyzed using a FACScalibur machine. The grey histograms represent isotype controls. (TIFF) [file pone.0056730.s002.tiff]

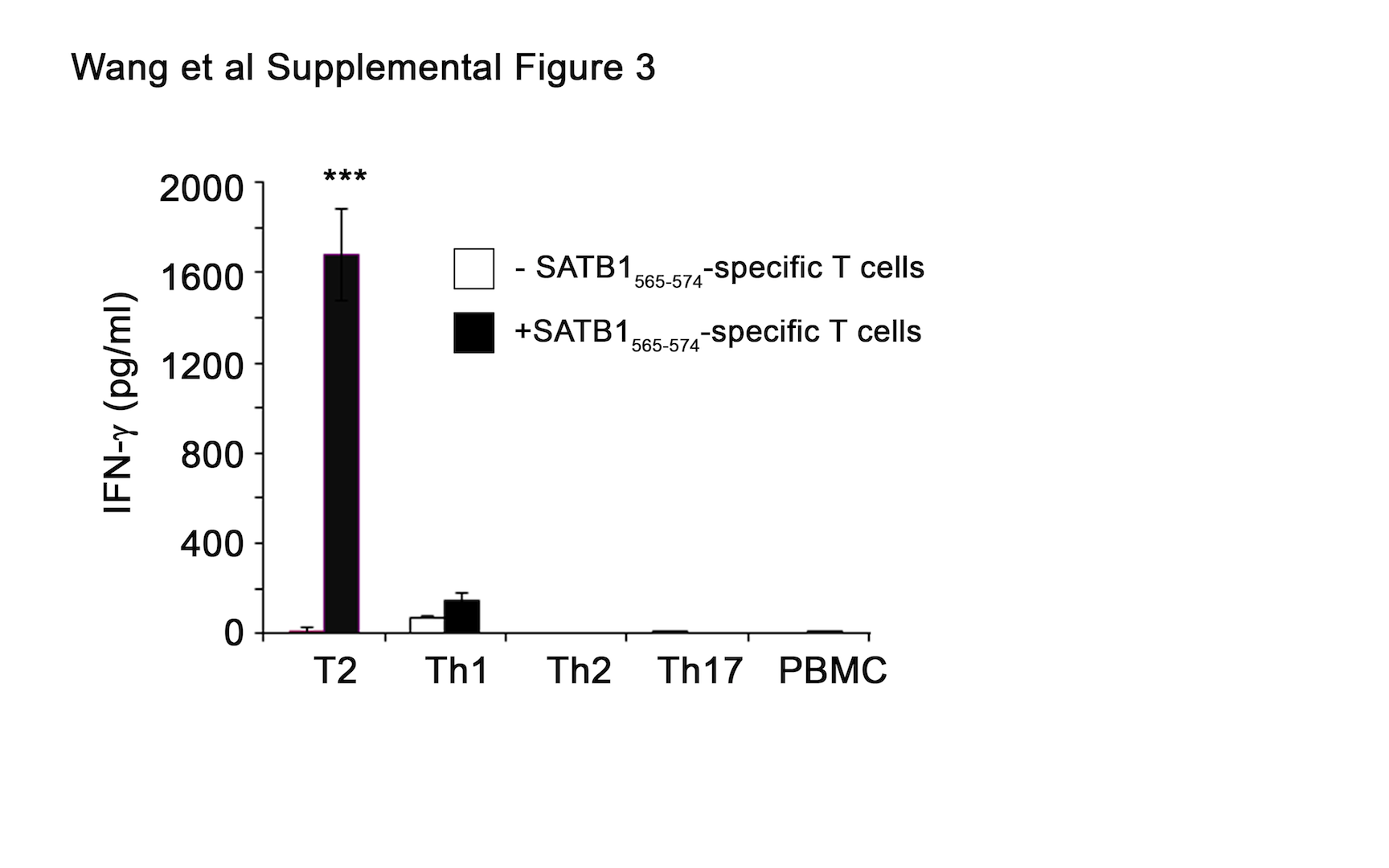

Supplement: Figure S3 — SATB1565–574 -specific T cells were not able to recognize in vitro-differentiated Th cell subsets. Different T cell subsets (Th1, Th2, Th17 and PBMC) were co-incubated without or with SATB1565–574 -specific CD8+ T cells (0.1×10^6^) in 96-well plate, respectively. Cells were incubated for 18–24 hours, the IFN-γ secretion in the supernatant was determined by ELISA assay. T2 cells loaded with SATB1565–574 were used as positive control. ***P<0.001 versus control (SATB1565–574–specific T cells stimulated with unloaded T2 cells). (TIFF) [file pone.0056730.s003.tiff]
