# Supplementary material for: Identification of Special AT-Rich Sequence Binding Protein 1 as a Novel Tumor Antigen Recognized by CD8+ T Cells: Implication for Cancer Immunotherapy
Source: PLoS One. 2013 Feb 21;8(2):e56730. doi: 10.1371/journal.pone.0056730 (PMC3578933; doi:10.1371/journal.pone.0056730)
Supplement: File S1 — Supplemental Materials and Methods. (DOCX) [file pone.0056730.s004.docx]

**Supporting Information File S1**

**Supplemental Materials and Methods**

**Reagents**

Human cytokines IL-12, IL-6, IL-23 and anti-human CD28 were purchased from R&D Systems (Minneapolis, MN, USA); Anti-CD3 (OKT3) was obtained from Ortho Biotech (Bridgewater, NJ, USA); IL-1β and anti-IFN-γ were obtained from Pierce Biotechnology (Rockford, IL, USA); Human recombinant IL-2 (hIL-2) was obtained from Chiron Corporation (Emeryville, CA, USA). IL-4 and IFN-γ were purchased from PeproTech (Rocky Hill, NJ, USA).

**Differentiation of T helper (Th) cell subsets**

Human CD4^+^ T cells (5x10^5^/mL) were stimulated with anti-CD3 (0.5 μg/mL) and anti-CD28 (1μg/mL) along with 2x10^6^ irradiated APC cells (50 Gy) for 6 days in the presence of different cytokine cocktails. For Th1 differentiation, a cytokine cocktail containing IL-12 (20 ng/mL) and hIL-2 (60 IU/mL) was used; For Th2 differentiation, a cytokine cocktail of IL-4 (20 ng/mL), hIL-2 (60 IU/mL) and anti-IFN-γ (5 μg/mL) was used; For induction of Th17 cells, a combination of IL-1β (10 ng/mL), IL-6 (20 ng/mL), IL-23 (20 ng/mL), IL-2 (60 IU/mL) and anti-IFN-γ (5μg/mL) was used. Cells were harvested at day 6 and used for ELISA assay.
